# Development of the Respiratory Index of Severity in Children (RISC) Score among Young Children with Respiratory Infections in South Africa

**DOI:** 10.1371/journal.pone.0027793

**Published:** 2012-01-04

**Authors:** Carrie Reed, Shabir A. Madhi, Keith P. Klugman, Locadiah Kuwanda, Justin R. Ortiz, Lyn Finelli, Alicia M. Fry

**Affiliations:** 1 Influenza Division, National Center for Immunization and Respiratory Diseases, Centers for Disease Control and Prevention, Atlanta, Georgia, United States of America; 2 Epidemic Intelligence Service, Office of Workforce and Career Development, Centers for Disease Control and Prevention, Atlanta, Georgia, United States of America; 3 Respiratory and Meningeal Pathogens Research Unit, Department of Science and Technology/National Research Foundation, Vaccine Preventable Diseases and Medical Research Council, University of the Witwatersrand, Bertsham, South Africa; 4 Hubert Department of Public Health, Rollins School of Public Health, Emory University, Atlanta, Georgia, United States of America; 5 University of Washington, Seattle, Washington, United States of America; Duke University School of Medicine, United States of America

## Abstract

**Objective:**

Pneumonia is a leading cause of death in children worldwide. A simple clinical score predicting the probability of death in a young child with lower respiratory tract infection (LRTI) could aid clinicians in case management and provide a standardized severity measure during epidemiologic studies.

**Methods:**

We analyzed 4,148 LRTI hospitalizations in children <24 months enrolled in a pneumococcal conjugate vaccine trial in South Africa from 1998–2001, to develop the Respiratory Index of Severity in Children (RISC). Using clinical data at admission, a multivariable logistic regression model for mortality was developed and statistically evaluated using bootstrap resampling techniques. Points were assigned to risk factors based on their coefficients in the multivariable model. A child's RISC score is the sum of points for each risk factor present. Separate models were developed for HIV-infected and non-infected children.

**Results:**

Significant risk factors for HIV-infected and non-infected children included low oxygen saturation, chest indrawing, wheezing, and refusal to feed. The models also included age and HIV clinical classification (for HIV-infected children) or weight-for-age (for non-infected children). RISC scores ranged up to 7 points for HIV-infected or 6 points for non-infected children and correlated with probability of death (0–47%, HIV-infected; 0–14%, non-infected). Final models showed good discrimination (area under the ROC curve) and calibration (goodness-of-fit).

**Conclusion:**

The RISC score incorporates a simple set of risk factors that accurately discriminate between young children based on their risk of death from LRTI, and may provide an objective means to quantify severity based on the risk of mortality.

## Introduction

Pneumonia is a major cause of morbidity and mortality in children less than age five years. The World Health Organization (WHO) estimates that lower respiratory tract infection (LRTI) is responsible for approximately 20% of deaths in children less than five years worldwide, 90% of which is pneumonia [1]. The highest mortality from childhood respiratory infection is seen in resource-limited countries and in children younger than 24 months of age [2]. In children with HIV infection, the burden of pneumonia is even greater, with a broader range of pathogens and a case-fatality ratio three- to six-times higher than children without HIV infection [3],[4].

Effective management of pneumonia in children includes an assessment of the severity of illness, and in many settings around the world, this may rely primarily on signs and symptoms ascertained at presentation. Case management in many resource-limited settings is based on the Integrated Management of Childhood Illness (IMCI) system developed by the World Health Organization to aid treatment decisions for major causes of death in children under five [5]. The IMCI classifies pneumonia, severe pneumonia, and very severe pneumonia with simple case definitions that include elevated respiratory rate, chest wall indrawing, and other danger signs. These deliberately sensitive definitions maximize the number of children identified who could benefit from antibiotic treatment or referral to a hospital. However, many countries report poor compliance with hospital referral due to geographic and socioeconomic constraints, undermining efforts to reduce childhood mortality [6], [7]. More specific discrimination of children with LRTI based on their risk of mortality may help refine decisions about case management, such as the most appropriate site of treatment or the need for additional supportive care.

Clinical prediction scores have been developed and validated to aid clinicians in managing and treating adult patients with community-acquired pneumonia [8], [9]. These scores assign points to a variety of patient characteristics, clinical signs and laboratory measurements to calculate a total score which objectively discriminates patients based on their risk of mortality [10]. No similar method has been developed to quantify the severity of pediatric pneumonia.

A simple pediatric severity score could compliment the current IMCI framework by predicting the probability of death in a child who presents with respiratory illness or provide a standardized means of quantifying severity among children during vaccine trials and other epidemiologic studies. The objective of this study was to develop a severity score for LRTI in children with and without HIV infection – the Respiratory Index of Severity in Children (RISC) – and assess its ability to predict mortality by evaluating the performance of the scoring model.

## Methods

### Ethics Statement

The original study was approved by the Committee for the Study of Human Subjects at the University of the Witwatersrand, and permission for the trial was obtained from the Medicines Control Council of South Africa. All participants were enrolled after written informed consent had been obtained from a parent or legal guardian. Secondary analysis of the original data for this manuscript was approved by the Institutional Review Board of the Centers for Disease Control and Prevention.

### Participants

We performed a secondary analysis of data from children <24 months of age who were enrolled in a clinical trial of the 9-valent pneumococcal conjugate vaccine in Soweto, South Africa and hospitalized with LRTI between 1998 and 2001. Details of the trial have been described elsewhere [11], [12]. Briefly, 39,836 children were randomized to receive 3 doses of the 9-valent pneumococcal conjugate vaccine or a placebo at 6, 10, and 14 weeks of age. All children also received *Haemophilus influenzae* type b (Hib) vaccine. Study staff monitored all admissions at the local hospital (Chris Hani-Baragwanath Hospital; Soweto, South Africa) through November 2001 and determined when study participants were admitted with LRTI (clinical diagnosis of pneumonia or bronchiolitis, irrespective of radiographic features). Children were tested for HIV infection when they were hospitalized for any reason. Details of HIV testing have been described previously [11].

All children hospitalized with LRTI during the follow-up period were potentially eligible for this analysis. Hospitalizations were excluded from the analysis if the child's HIV infection status was unknown, data on illness history or physical exam was not collected, or the child was ≥24 months at the time of admission. Children who received the study vaccine were less likely to be hospitalized with respiratory illness than those in the comparison arm [11], but among those hospitalized with LRTI, vaccinated children did not have reduced mortality compared with unvaccinated children. Consequently, children in both the vaccine and comparison arms were included in this analysis.

### Measures

The outcome of interest was in-hospital mortality among children hospitalized with LRTI. As potential predictors of mortality, we considered the following classes of variables: Demographics, medical history, history of present illness, signs on physical exam, growth standards, chest radiography, and C-reactive protein levels. Information on these variables was collected by study physicians on a standardized case report form when a child was hospitalized. Subjective information on symptoms occurring prior to hospitalization was obtained from the child's caregiver at the time of hospitalization.

For this analysis, age was categorized based on IMCI categories: 6 weeks–2 months, 3–12 months, and 12–23 months. Children were considered to have low oxygen saturation if a pulse oximetry reading on room air was ≤90%. Three growth standards were also evaluated: weight for age, weight for length, and length for age, categorized based on the WHO z-scores [13]. Tables of growth standards were accessed at: http://www.who.int/childgrowth/standards/. Chest radiographs were evaluated independently by a pediatrician and a radiologist. C-reactive protein levels were categorized as >40 mg/L or ≤40 mg/L, which may indicate bacterial pneumonia [14]. For children with HIV infection, the clinical classification of HIV disease without CD4 count was recorded using the CDC categories – N (asymptomatic), A (mildly symptomatic), B (moderately symptomatic), C (severely symptomatic, AIDS-defining) [15].

### Impact of HIV status

Because of the high prevalence of HIV infection in the study population and the substantial impact of HIV infection on mortality in young children, separate models were developed for HIV-infected and non-infected children. The cohort was stratified by HIV status and the following methods were used first to develop a model for children without HIV infection, and then repeated in the subset of children with HIV infection.

According to the local standard of care at the time, antiretroviral drugs for the treatment of HIV infection were not routinely used. Trimethoprim-sulfamethoxazole prophylaxis was recommended for HIV-exposed children aged 6 weeks or as soon as the child received a diagnosis of HIV infection if prophylaxis was not previously started; prophylaxis continued past the age of 1 year in children with AIDS of clinical category B or C [16].

### Identifying risk factors for mortality

Using mortality as a dichotomous outcome variable, we assessed the relationship between each risk factor and mortality. Odds ratios for mortality and their 95% confidence intervals, as well as p-values, were calculated for each risk factor using logistic regression with generalized estimating equations (GEE) to account for the inclusion of multiple episodes among some children. Variables with p<0.20 in univariate analysis were considered as candidate predictors for multivariable regression. To develop the multivariable model, we evaluated included variables for interaction, and then all candidate variables were included in a GEE model and non-significant variables were eliminated one at a time, starting with the variable with the smallest magnitude of effect, until all remaining variables had p<0.05 or removing an additional variable significantly increased the −2 log likelihood of the model.

When developing the multivariable model, we initially chose to exclude some variables that, although associated with mortality, could have reduced the usability of the index in some settings, including chest radiography and laboratory measurement of CRP levels. After selecting a multivariable model, each of these variables was then added to assess whether they improved the predictive performance of the model.

### Developing a scoring system

A point-based scoring system was developed from the final multivariable logistic regression model in which a number of points was assigned to each predictor in the model by rounding each β coefficient to the nearest integer. For consecutive cut-offs of the summed scores, the sensitivity, specificity and positive predictive values for mortality were calculated.

### Statistical validation

To assess the predictive accuracy of our scoring system, two measures of model performance were evaluated – discrimination and calibration. Models with good discrimination distinguish well between patients with and without the outcome of interest. Discrimination was measured using the c-statistic, or area under the receiver operating characteristic (ROC) curve. The c-statistic ranges from 0.5 to 1, with higher values indicating better discrimination. Calibration measures how well the predicted probabilities of death match the actual mortality observed across the data. Calibration was assessed using the Hosmer-Lemeshow goodness of fit test. A low chi-square and high p-value indicates good calibration. Calibration was also assessed visually by graphing the observed frequencies of mortality against the predicted probabilities of mortality at each score level.

The performance of a prediction model is generally worse in new patients than initially observed when developing the model. This ‘optimism’ can be assessed with standard bootstrapping procedures [17]. To assess the internal validity of our model, bootstrap samples were drawn with replacement and of the same size as the original sample. The multivariable model was re-estimated in 300 bootstrap samples and each model was evaluated on the new sample and the original data. The average difference in the c-statistic indicates the optimism in the initially estimated discrimination [18].

All statistical analysis was performed using SAS software version 9.2 (SAS Institute, Cary, NC).

## Results

### Study population

From 1998 to 2001, 4,584 hospitalizations with LRTI were observed in 3,140 children enrolled in the 9-valent pneumococcal conjugate vaccine trial in South Africa. Episodes of LRTI were excluded from the present study if they had unknown HIV status (n = 101, 2.2%), if either illness history (n = 21, 0.5%) or physical exam (n = 23, 0.5%) were not assessed, or the child was ≥24 months of age at the time of hospitalization (n = 319, 6.9%). Overall, 4,148 (90.5%) LRTI hospitalizations observed during the vaccine trial were included in this study.

Thirty-six percent (n = 1,502) of all LRTI hospitalizations occurred among children with HIV infection. Mortality varied greatly by HIV status, as 17.6% of all episodes in children with HIV infection (n = 265) resulted in death, compared to 1.3% of all episodes in children without HIV infection (n = 33).

### Scoring model

Risk factors associated with mortality were identified for children with and without HIV infection (see Table 1). In multivariable regression (Table 2), independent risk factors for mortality in HIV non-infected children included: low oxygen saturation on room air, chest wall indrawing, low weight for age, and refusal to feed. Wheezing was associated with a decreased risk of mortality. Independent risk factors for mortality in HIV-infected children were slightly different and included: low oxygen saturation on room air, chest wall indrawing, and refusal to feed, but also age, and HIV clinical classification. Wheezing was also associated with a decreased risk of mortality in HIV-infected children. Due to interaction between oxygen saturation and chest indrawing in the multivariable model, we combined these factors in the final model; chest indrawing was only associated with increased mortality if oxygen saturation was normal.

**Table 1:** Description and univariate analysis of risk factors for mortality among HIV non-infected and HIV-infected children <24 months of age hospitalized with LRTI.

	HIV non-infected			HIV-infected		
	% of total	% of deaths	OR (95%CI)	% of total	% of deaths	OR (95%CI)
	n = 2,646	n = 33		n = 1,502	n = 265	
**Patient history**						
Age						
0–2 months	18.0	27.3	3.0 (0.9–9.9)*	16.8	30.9	5.5 (3.4–9.1)**
3–12 months	58.0	60.6	2.1 (0.7–6.1)*	63.2	60.4	2.4 (1.5–3.7)**
13–23 months	24.0	12.1	1.0 (Ref)	20.0	8.7	1.0 (Ref)
Male sex	58.9	51.2	0.7 (0.4–1.5)	53.9	49.2	0.8 (0.6–1.0)*
Premature	21.0	36.4	2.2 (1.1–4.5)**	20.0	18.9	0.9 (0.6–1.3)
Received study vaccine	49.3	45.5	0.9 (0.4–1.7)	45.6	49.4	1.2 (0.9–1.6)
CDC HIV clinical classification	n/a	n/a	n/a			
Severe (C)				35.0	55.3	8.1 (4.1–16.2)**
Mild/moderate (A/B)				54.2	41.6	3.1 (1.6–6.1)**
Asymptomatic (N)				10.8	3.1	1.0 (Ref)
**History of present illness**						
Had diarrhea	20.3	28.1	1.5 (0.7–3.4)	32.2	36.7	1.2 (1.0–1.6)*
Had vomiting	42.8	39.4	0.9 (0.4–1.7)	42.5	40.8	0.9 (0.7–1.2)
Irritable/excessive crying	37.4	28.1	0.7 (0.3–1.4)	47.6	51.7	1.2 (0.9–1.5)*
Refusing feeds	37.8	51.5	1.7 (0.9–3.4)*	41.8	50.2	1.5 (1.2–1.9)**
Seizures	3.4	9.4	3.0 (0.9–9.8)*	1.9	2.7	1.7 (0.8–3.6)
**Physical exam**						
Abnormal temperature	27.9	27.3	0.9 (0.4–2.5)	33.9	32.8	0.9 (0.7–1.3)
O_2_ saturation on room air <90%	27.5	84.9	15.2 (5.9–39.4)**	56.2	84.5	5.6 (4.0–7.7)**
Chest indrawing	39.8	81.8	7.0 (2.9–16.9)**	65.0	84.2	3.3 (2.4–4.5)**
Intercostal recession	66.3	93.9	8.0 (1.9–33.1)**	85.1	92.5	2.4 (1.5–3.6)**
Wheezing at exam	55.0	9.1	0.1 (0.02–0.3)**	16.6	7.6	0.4 (0.3–0.6)**
Elevated respiratory rate, for age	56.9	75.8	2.4 (1.1–5.3)**	70.7	81.7	2.1 (1.5–2.9)**
>20 breaths per minute	9.7	30.0	4.2 (2.0–8.9)**	22.8	35.9	2.2 (1.6–2.8)**
Crepitations	53.4	75.0	2.6 (1.2–5.9)**	61.8	61.2	1.0 (0.8–1.3)
Bronchial breathing	3.5	15.6	5.4 (2.0–14.2)**	14.8	17.6	1.4 (0.9–1.9)*
Pulse rate >170	9.3	9.1	0.9 (0.3–3.2)	13.2	17.2	1.4 (1.0–2.0)**
Weight for age						
Low (−3<z-score≤−2)	7.9	15.2	4.8 (1.7–13.9)**	20.1	18.5	1.1 (0.7–1.5)
Very Low (z-score≤−3)	8.8	48.5	14.5 (6.6–31.6)**	37.5	44.2	1.5 (1.1–2.0)**
Length for age						
Low (−3<z-score≤−2)	12.0	22.6	2.7 (1.1–6.5)**	16.7	15.0	0.9 (0.6–1.3)
Very Low (z-score≤−3)	15.8	25.8	2.3 (1.0–5.5)**	37.1	38.6	1.1 (0.8–1.4)
Weight for length						
Low (−3<z-score≤−2)	7.1	16.1	3.5 (1.3–9.5)**	15.1	14.7	1.1 (0.7–1.5)
Very Low (z-score≤−3)	5.2	25.8	7.9 (3.3–18.6)**	23.7	27.5	1.3 (1.0–1.8)**
**Radiology & Laboratory**						
Alveolar consolidation on x-ray†	16.5	52.6	4.5 (2.1–9.9)**	37.5	35.8	1.1 (0.8–1.5)
Other infiltrates on x-ray†	23.4	10.5	0.8 (0.3–2.1)	22.7	18.7	0.6 (0.4–0.9)**
CRP≥40 mg/L†	24.8	45.5	1.8 (0.9–3.7)*	34.5	34.0	0.9 (0.7–1.2)

*p<0.20;

**p<0.05.

† *Missing observations for >10%;*

**Table 2:** Independent risk factors for mortality in children <24 months hospitalized with LRTI, by HIV status.

	aOR	95%CI	p-value	Score
**HIV non-infected**				
Most severe respiratory sign:				
O_2_ saturation<90%	20.9	(5.0–87)	<0.01	3
Chest indrawing	4.6	(2.2–9.4)	<0.01	2
Wheezing	0.2	(0.05–0.6)	<0.01	−2
Refusing feeds	1.8	(0.9–3.8)	0.10	1
Weight for age:				
Low (≤−2 z-score)	2.5	(1.6–3.8)	<0.01	1
Very low (≤−3 z-score)	6.0	(2.5–14.4)	<0.01	2
**HIV-infected**				
Most severe respiratory sign:				
O_2_ saturation<90%	4.8	(3.0–7.6)	<0.01	2
Chest indrawing	2.2	(1.7–2.8)	<0.01	1
Wheezing	0.6	(0.4–0.9)	0.03	−1
Refusing feeds	1.5	(1.2–2.0)	<0.01	1
HIV classification				
Severe (C)	5.5	(2.5–12.1)	<0.01	2
Mild or moderate (A/B)	2.3	(1.1–5.0)	0.02	1
Not symptomatic (N)	1.0	-	-	-
IMCI age group				
≤2 months	6.0	(3.5–10.4)	<0.01	2
3–12 months	2.2	(1.4–3.4)	<0.01	1
13–23 months	1.0	-	-	-

A number of points was assigned to each of the risk factors in the final models and is shown in Table 3. An overall score was calculated for each episode of LRTI by adding the points for each risk factor identified at presentation. For example, a child without HIV infection who presented with low oxygen saturation (3 points) and was wheezing (subtract 2 points), but was of normal weight for age (0 points) and was not refusing feedings (0 points) would have a total RISC score of 1 point.

**Table 3:** RISC scoring system.

HIV NON-INFECTED CHILDREN		
**Severity of respiratory signs on physical exam:**	**If O_2_≤90%:**	3 points
**1. Oxygen saturation = _____%**	**else**	
**2. Does the child have chest indrawing?** Yes/No	**Indrawing:**	2 points
**3. Does the child have wheezing?** Yes/No	**Wheezing:**	−2 points
**4. Has the child been refusing feedings?** Yes/No	**Refusal to feed:**	1 point
**Growth standards:**		
**5. Weight for age z-score = ______**	**z≤−3:**	2 points
Weight = _____ kg Age = ______ months	**−2≤z<−3:**	1 points
	**z>−2:**	0 points
	**Total points:**	_______
	(Maximum:	6)

HIV-INFECTED CHILDREN		
**Severity of respiratory signs on physical exam:**	**If O_2_≤90%:**	2 points
**1. Oxygen saturation = _____%**	**else**	
**2. Does the child have chest indrawing?** Yes/No	**Indrawing:**	1 point
**3. Does the child have wheezing?** Yes/No	**Wheezing:**	−1 points
**4. Has the child been refusing feedings?** Yes/No	**Refusal to feed:**	1 point
	**0–2 months:**	2 points
**5. Age** = _____ months	**3–12 months:**	1 points
	**>12 months:**	0 points
	**C:**	2 points
**6. HIV Clinical Classification** = N/A/B/C	**A/B:**	1 points
	**N:**	0 points
	**Total points:**	_______
	(Maximum:	7)

**Figure 1:**
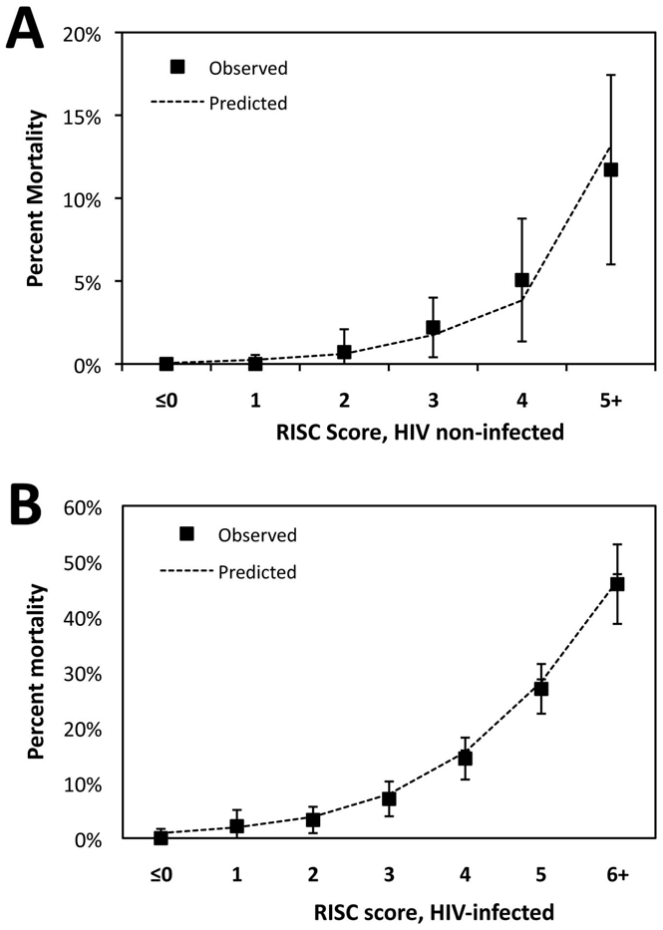
Observed mortality (with 95% confidence intervals) and mean predicted mortality by RISC score for children <24 months hospitalized with LRTI, (A) without HIV infection and (B) with HIV infection.

The RISC scores for children ranged up to 7 points for HIV-infected children and 6 points for non-infected children. For each score level, the observed risk of mortality and predicted probability of death from the model is shown in Figure 1. Among HIV non-infected children in this population, the median RISC score was 1 (Table 4), with a corresponding risk of mortality of 0% (95%CI: 0%–0.6%) (Figure 1). Among HIV-infected children, with a higher overall risk of mortality, the median RISC score was 4 (Table 4), with a mortality of 14.4% (95%CI: 10.6%–18.2%).

**Table 4:** Distribution of RISC scores and screening performance in children <24 months of age with and without HIV infection, hospitalized with LRTI in Soweto, South Africa 1998–2001.

Score	Percent of total	Percent Mortality (95%CI)	Sensitivity*	Specificity*	Positive Predictive Value*	Negative Predictive Value*
**HIV−**						
**≤0**	46.7	0.0 (0–0.2)	100%	0%	1%	n/a
**1**	19.4	0.0 (0–0.6)	100%	48%	2%	100%
**2**	10.7	0.7 (0–1.7)	100%	67%	4%	100%
**3**	12.2	2.2 (0.6–3.8)	94%	78%	5%	100%
**4**	6.1	5.1 (1.6–8.5)	72%	90%	8%	100%
**5**	3.5	10.9 (4.5–17.2)	47%	96%	12%	99%
**6**	1.4	13.9 (2.6–25.2)	16%	99%	13%	99%
**HIV+**						
**≤0**	2.4	0.0 (0–1.7)	100%	0%	17%	n/a
**1**	6.3	2.2 (0–5.1)	100%	3%	18%	100%
**2**	14.5	3.3 (0.9–5.7)	99%	10%	19%	98%
**3**	17.2	7.1 (3.9–10.2)	97%	28%	22%	97%
**4**	22.2	14.4 (10.6–18.2)	90%	50%	27%	96%
**5**	25.2	27.0 (22.4–31.5)	71%	73%	35%	92%
**6**	11.0	45.7 (38.0–53.3)	32%	93%	48%	87%
**7**	1.3	47.4 (24.9–69.8)	4%	99%	53%	83%

*For mortality, using a cutoff at the corresponding score value.

We considered three variables as additions to the multivariable model for HIV non-infected children to determine whether they improved the performance of the final model. In multivariable analysis, hepatomegaly (aOR = 3.1, 95%CI: 1.4–6.8) or the presence of alveolar consolidation on chest radiograph (aOR = 2.0, 95%CI: 0.9–4.7) were each independently associated with mortality in HIV non-infected children, though the discrimination of the multivariable model was already high and did not improve with either addition. In addition, 198 (7.5%) episodes would have been excluded due to missing data on chest radiograph. Elevated CRP level was not a statistically significant independent predictor of mortality in children without HIV infection (OR = 1.6, 95%CI: 0.7–3.7), and excluded 908 (34.3%) episodes. Thus, these variables were not retained in the final RISC score, but may warrant further consideration.

### Validation

**Figure 2:**
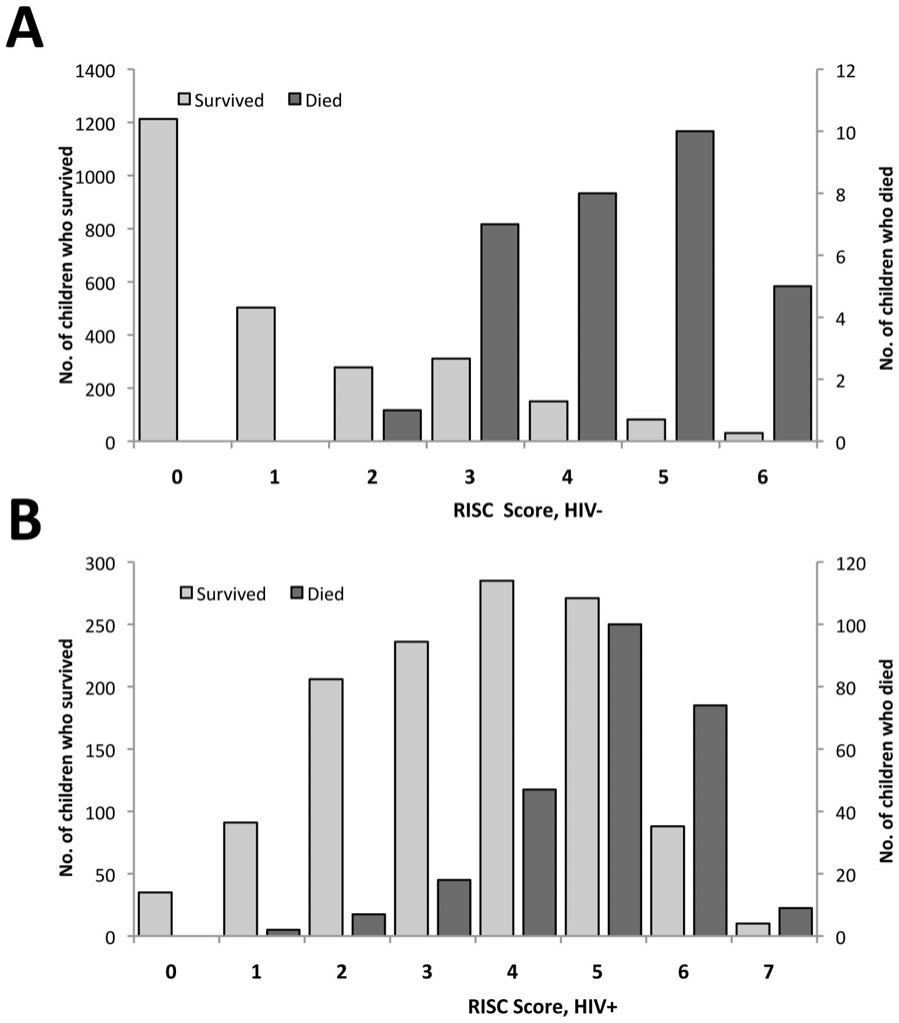
Distribution of RISC score stratified by mortality for children <24 months hospitalized with LRTI, (A) without HIV infection and (B) with HIV infection.

The discrimination and calibration for both the HIV-infected and non-infected models were measured to evaluate the performance of the RISC score. The c-statistic, or area under the ROC curve, is a measure of model discrimination and ranges from 0.5 to 1, with higher values indicating better discrimination. In the full dataset, the models showed good discrimination (c-statistic = 0.776 for HIV-infected and 0.923 for non-infected children) and calibration (p-value for goodness-of-fit = 0.95 for HIV-infected and 0.87 for non-infected children), although the model for HIV non-infected children provided better discrimination of mortality than the model for HIV-infected children (see Table 4 and Figure 2). We also evaluated the internal validity of the RISC model using standard bootstrapping techniques to estimate the optimism, or possible over-fitting, in our measurement of model discrimination. From 300 bootstrap samples, the c-statistic from models among HIV non-infected children ranged from 0.742–0.926, with an estimated average optimism of 0.013 in the original data. For HIV-infected children, the c-statistic from models of 300 bootstrap samples ranged from 0.665–0.732, with an estimated average optimism of 0.004.

## Discussion

We developed and validated a clinical prediction score, the RISC score, which uses a simple set of clinical factors to discriminate between young children with varying risks of death from LRTI. Variables retained in the score represent known risk factors for severe outcomes of respiratory illness in children, including: hypoxemia, chest indrawing, refusal to feed, malnutrition, age, and stage of HIV disease [19], [20], [21], [22], [23], [24], [25]. We demonstrate that a simple combination of these factors in a scoring model provides good discrimination and calibration for predicting mortality in young children with LRTI, without the need for radiographic or laboratory measurements.

This severity index has several potential uses in both research and clinical settings. In the research setting this tool may provide a useful means of quantifying the severity of LRTI in epidemiologic studies and clinical trials. In clinical settings, the ability to quantify the risk of mortality may aid clinicians in the management of young children that present with LRTI and compliment current IMCI guidelines. Recent studies have indicated that some children with severe pneumonia by IMCI criteria may be successfully managed at home [26], and that in regions with barriers to hospital referral, limiting referrals to those most in need actually improved the overall number of children who received appropriate treatment and may have reduced mortality [6]. The use of the RISC score may help refine decisions about case management in resource-limited countries or areas with geographic and socioeconomic barriers to hospital referral by facilitating decisions about the most appropriate site of treatment (i.e., home vs. hospital) or the need for additional supportive care (i.e., supplemental oxygen or intensive care).

Hypoxemia was an important risk factor among both HIV-infected and non-infected children. While pulse oximeters may not be currently available in all settings, our finding supports several recent studies that have shown the importance of pulse oximetry in guiding the use of oxygen therapy and other supportive care for reducing the mortality in children with respiratory infection [27], [28]. In contrast, wheezing was associated with reduced mortality in both models. There have been concerns about the potential for misclassifying pneumonia in children with wheeze, which can also result in rapid breathing and thus an IMCI classification of pneumonia. Studies of children with IMCI-defined pneumonia and wheeze have found deterioration was more likely in children with additional danger signs such as chest indrawing or malnutrition [29]. In the absence of additional danger signs, children with wheeze would have a low RISC score, which supports suggestions that in many instances these young children might be successfully managed without hospitalization [29].

Predictive models are expected to perform better in the population in which they were developed than in other populations [30]. We used bootstrapping techniques to estimate the possible optimism associated with the apparent performance of the RISC model in this population. However, while the RISC score includes recognized predictors of severe LRTI, differences in the distribution of risk factors and/or health seeking behaviors in different populations may impact the performance of this model at predicting the risk of death, and will need to be evaluated. Efforts are ongoing to validate these scoring models in other populations of young children with respiratory illness to further define the predicted mortality across the RISC score in a variety of settings. Further study should also focus on evaluating the usefulness and applicability of this tool in different settings [31].

For children with HIV infection, this study was conducted prior to antiretroviral medication use in South Africa. Antiretroviral medication programs can reduce the incidence and severity of HIV-associated pneumonia in children [32], and the impact on this score is unclear. This highlights the need for further evaluation of this model in other populations to determine how the RISC score may best be applied or refined in other contexts.

In order to develop a score that would have high utility in resource-limited settings, which have the greatest burden of childhood mortality from LRTI, we considered the effect of radiographic and laboratory measurements only after developing a strong multivariable model. In this population, these variables did not provide any additional discrimination after other risk factors in the multivariable model and thus was not included in the final RISC score, but may warrant future consideration.

Finally, these data come from a cohort of children enrolled in a pneumococcal vaccine trial. Although there were no differences in mortality between children hospitalized with LRTI in either the vaccine or comparison group, half of the children did receive pneumococcal conjugate vaccine, and all received Hib vaccine. Additionally, although study investigators passively monitored hospitalizations with LRTI and did not directly participate in their clinical care, mortality observed in a vaccine trial may underestimate the risk of mortality that would exist in populations with less access to care. This underscores the need for further study of RISC in additional populations, to understand how it might be adapted in different settings.

The RISC score incorporates a simple set of variables that discriminate the probability of death in children hospitalized with LRTI. The ability to estimate a child's risk of mortality with limited clinical information may provide the ability to improve outcomes for children in resource-limited settings where the burden of pediatric pneumonia is highest. With further validation in additional populations, the RISC score may be a tool to more effectively manage LRTI, a major cause of death in children worldwide, and to better understand the impact of vaccination or other public health interventions on the severity of childhood respiratory illness.
